# Effects and Action Mechanisms of Berberine and *Rhizoma coptidis* on Gut Microbes and Obesity in High-Fat Diet-Fed C57BL/6J Mice

**DOI:** 10.1371/journal.pone.0024520

**Published:** 2011-09-06

**Authors:** Weidong Xie, Dayong Gu, Jianna Li, Kai Cui, Yaou Zhang

**Affiliations:** 1 Life Science Division, Graduate School at Shenzhen, Tsinghua University, Shenzhen, China; 2 Institute of Disease Control and Prevention, Shenzhen International Travel Health Care Center, Shenzhen Entry-Exit Inspection and Quarantine Bureau, Shenzhen, China; Institut de Pharmacologie et de Biologie Structurale, France

## Abstract

Gut microbes play important roles in regulating fat storage and metabolism. *Rhizoma coptidis* (RC) and its main active compound, berberine, have either antimicrobial or anti-obesity activities. In the present study, we hypothesize that RC exerts anti-obesity effects that are likely mediated by mechanisms of regulating gut microbes and berberine may be a key compound of RC. Gut microbes and glucose and lipid metabolism in high-fat diet-fed C57BL/6J (HFD) mice in vivo are investigated after RC and berberine treatments. The results show that RC (200 mg/kg) and berberine (200 mg/kg) significantly lower both body and visceral adipose weights, and reduce blood glucose and lipid levels, and decrease degradation of dietary polysaccharides in HFD mice. Both RC and berberine significantly reduce the proportions of fecal Firmicutes and Bacteroidetes to total bacteria in HFD mice. In the trial ex vivo, both RC and berberine significantly inhibit the growth of gut bacteria under aerobic and anaerobic conditions. In in vitro trials, both RC and berberine significantly inhibit the growth of *Lactobacillus* (a classical type of Firmicutes) under anaerobic conditions. Furthermore, both RC and berberine significantly increase fasting-induced adipose factor (Fiaf, a key protein negatively regulated by intestinal microbes) expressions in either intestinal or visceral adipose tissues. Both RC and berberine significantly increase mRNA expressions of AMPK, PGC1α, UCP2, CPT1α, and Hadhb related to mitochondrial energy metabolism, which may be driven by increased Fiaf expression. These results firstly suggest that antimicrobial activities of RC and berberine may result in decreasing degradation of dietary polysaccharides, lowering potential calorie intake, and then systemically activating Fiaf protein and related gene expressions of mitochondrial energy metabolism in visceral adipose tissues. Taken together, these action mechanisms may contribute to significant anti-obesity effects. Findings in the present study also indicate that pharmacological regulation on gut microbes can develop an anti-obesity strategy.

## Introduction

Obesity has emerged worldwide since 1999 with a stable and increasing tendency [1]. Obesity has profound impacts on cardiovascular diseases. However, the prevention and treatment of obesity remain significant challenges to medicinal scientists [2]. Obesity is derived from interactions of genetic and environmental factors. Gut microbes play important roles in regulating fat storage and metabolism [3]. Although gut microbes are potential targets, no appropriate drugs that target them have been developed thus far.

In traditional Chinese medicine, *Rhizoma coptidis* (RC, containing the active component berberine) is mostly used to clear heat, purge sthenic fire, and eliminate toxic materials. RC and berberine affect a variety of organisms, including bacteria, viruses, and fungi [4]–[9]. In China, RC and berberine are mostly used as drugs against intestinal infections [10]. RC and berberine have significant effects in lowering blood glucose, lipid, and body weight [11]–[15], and most studies indicate that RC and berberine directly exert metabolism-regulating effects [11]–[13]. However, berberine has low bioavailability after oral administration. Actually, it may be very difficult to reach effective concentrations in metabolism-regulating tissues in vivo, eg. visceral adipose tissues. Pharmacokinetics studies show that the maximum plasma concentrations of berberine vary from 0.01 to 0.40 µg/ml and even are lower than 0.01 µg/ml after oral administration [16]. The actual concentrations of berberine in visceral adipose tissues may be far lower than that in blood since these tissues have limited blood flow. However, studies in vitro indicate that the effective concentrations of berberine vary from 1 to 10 µg/ml [17], [18]. So, there are concerns about whether berberine can completely exert the effects after its limited intestinal absorption. It seems to be more reasonable to conclude that effects of berberine or RC contained berberine might mediate by targeting on gut tissues.

Taken together, in the present study, we hypothesize that RC exerts anti-obesity effects partly mediated by mechanisms of regulating gut microbes, and berberine may be a key active compound of RC that is responsible for these effects. The effects of RC and berberine in gut microbes, obesity, and their relationships are investigated in high-fat diet-fed C57BL/6J (HFD) mice.

## Results

### Effect of RC and berberine on body weight, visceral adipose tissues, and metabolic parameters in high-fat diet-fed C57BL/6J mice

**Figure 1:**
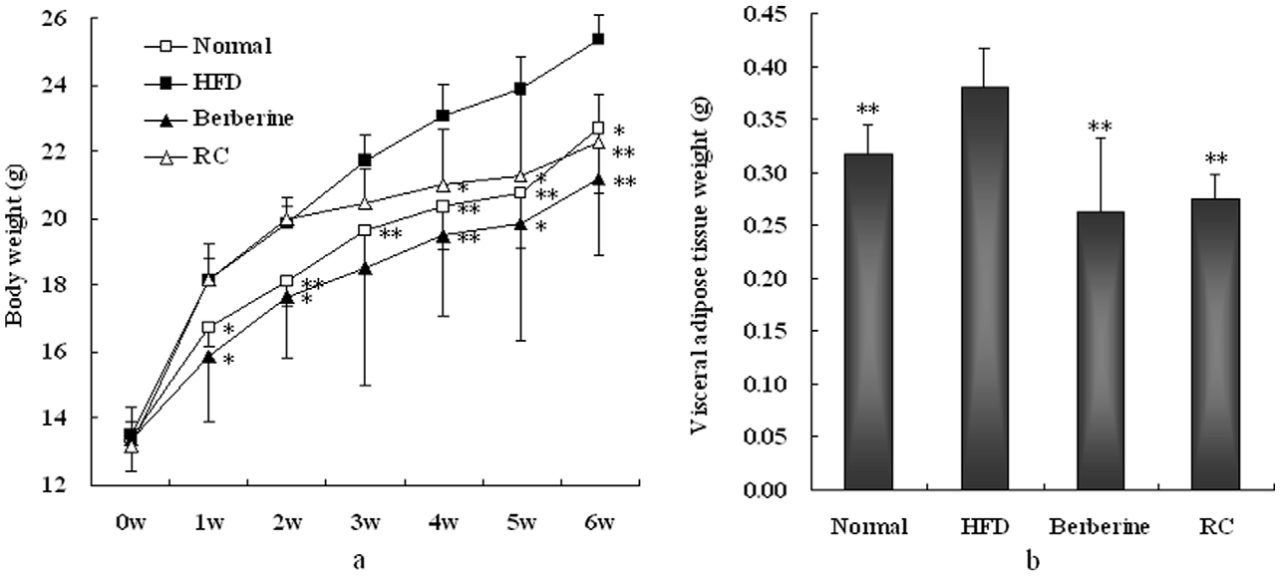
Effects of RC and berberine on (a) body weight and (b) visceral adipose tissue weight in HFD mice. “Normal”, normal chow diet-fed mice; “HFD”, high-fat diet-fed control mice; “berberine”, berberine-treated HFD mice; “RC”, *Rhizoma coptidis*-treated HFD mice. Data were expressed as Mean±S.D. (n = 10), *P<0.05, **P<0.01 vs. HFD controls.

Compared with normal chow diet-fed controls, HFD mice showed significant increases in body and visceral adipose weights (Fig. 1). However, RC and berberine significantly inhibited increases in body and fat weights in HFD mice. In trials of metabolic cages, HFD mice showed increased calorie intakes compared with normal controls (Table 1), which may be why HFD showed increases in body weight gain. RC and berberine did not significantly affect food intake, which suggests that they exert anti-obesity effects that are completely independent of food intake. In addition, HFD mice increased water intake and urine excretion, but decreased fecal excretion compared with normal controls. RC and berberine significantly decreased water intake and showed a decreased tendency to excrete urine, although not in a significant manner. Furthermore, HFD mice showed decreased fecal excretion compared with normal controls, but RC and berberine normalized this decrease.

**Table 1:** Effects of RC and berberine on blood biochemical assay parameters, metabolic cage trials, and fecal lipid and polysaccharide evaluations in HFD mice.

	Normal	HFD	Berberine	RC
Blood glucose (mmol/L)	7.1±0.7*	8.2±0.8	6.5±1.5*	5.8±1.2**
Serum total cholesterol (mg/dl)	98.7±8.0**	120.9±8.7	100.3±15.7*	96.6±11.8*
serum triglycerides (mmol/L)	0.97±0.21	1.05±0.06	1.02±0.43	0.57±0.08**
Diet intake (Kcal/mouse)	11.2±0.59**	13.1±1.49	11.9±2.00	13.7±2.13
Water intake (ml/mouse)	3.0±1.09**	4.3±0.54	3.9±0.29*	3.7±0.27**
Fecal excretion (g/mouse)	1.6±0.39**	0.9±0.23	1.6±0.29**	1.3±0.13**
Urine excretion (ml/mouse)	1.3±0.54*	2.0±0.67	1.8±0.40	1.7±0.33
Fecal triglycerides (mg/g)	2.16±0.41**	3.45±0.88	2.90±0.69	2.86±0.53
Fecal cholesterol (mg/g)	3.81±1.09**	8.37±1.71	6.62±1.29*	6.53±1.40*
Fecal polysaccharides (mg/g)	16.4±7.2	18.5±3.1	35.3±6.3**	33.4±2.4**

“Normal”, normal chow diet-fed mice; “HFD”, high-fat diet-fed control mice; “berberine”, berberine-treated HFD mice; “RC”, *Rhizoma coptidis*-treated HFD mice. Data were expressed as Mean±S.D. (n = 10),

*P<0.05,

**P<0.01 vs. HFD controls.

### Effect of RC and berberine on blood glucose, lipid, and fecal lipid and polysaccharide levels in high-fat diet-fed C57BL/6J mice

HFD mice showed significant increases in blood glucose and total cholesterol but had no significant change in serum triglycerides compared with normal controls (Table 1). RC and berberine significantly inhibited increases in blood glucose and total cholesterol levels in HFD mice. In addition, RC significantly lowered serum triglycerides in HFD mice, whereas berberine did not. For fecal lipid assays, HFD mice showed significant increases in fecal triglycerides and total cholesterol levels compared with normal controls. RC and berberine significantly decreased fecal cholesterol concentrations and showed decreasing tendencies of fecal triglycerides but not in a significant manner. This suggests that both RC and berberine have anti-obesity activities or lipid-lowering effects, but not by reducing intestinal lipid absorption. By estimation, the caloric content of fecal lipid excretion was less than 1% of the total dietary caloric intake and negligible in all groups. It can be concluded that both RC and berberine may exert anti-obesity activities or lipid-lowering effects through enhanced energy expenditure. In addition, HFD mice did not show significant change of fecal polysaccharide content compared with normal controls. Interestingly, both RC and berberine significantly increased the fecal polysaccharide content. Therefore, RC and berberine exert anti-obesity activities or lipid-lowering effects partly by mechanisms of inhibiting degradation of dietary polysaccharides.

### Effect of RC and berberine on 16SrRNA gene of Bacteroidetes and Firmicutes in feces of high-fat diet-fed C57BL/6J mice

**Figure 2:**
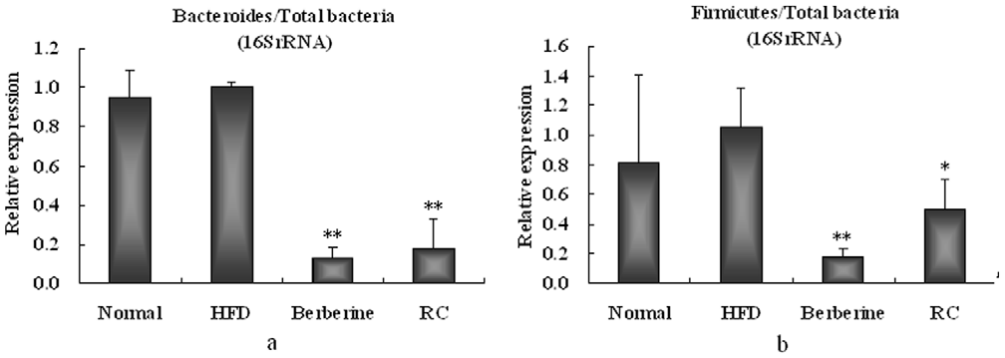
Effects of RC and berberine on 16SrRNA gene relative expressions of (a) Bacteroides/total bacteria and (b) Firmicutes/total bacteria in feces of HFD mice. “Normal”, normal chow diet-fed mice; “HFD”, high-fat diet-fed control mice; “berberine”, berberine-treated HFD mice; “RC”, *Rhizoma coptidis*-treated HFD mice. Data were expressed as Mean±S.D. (n = 6), *P<0.05, **P<0.01 vs. HFD controls.

Six mice from each group were randomly selected for the trials. Gene expressions of 16SrRNA showed that there were no significant differences in the ratios of fecal Firmicutes or Bacteroidetes to total bacteria between HFD and normal mice, although the ratio of Firmicutes to total bacteria showed increased tendencies in HFD mice compared with normal controls (Fig. 2). RC and berberine significantly inhibited the ratios of Firmicutes or Bacteroidetes to total bacteria in the feces of HFD mice. RC and berberine significantly reduced Bacteroidetes compared with HFD controls by 81.8% and 87.4%, respectively, and Firmicutes compared with HFD controls by 49.7% and 82.4%, respectively.

### Effect of RC and berberine on growth of fecal bacteria ex vivo under aerobic and anaerobic conditions

**Figure 3:**
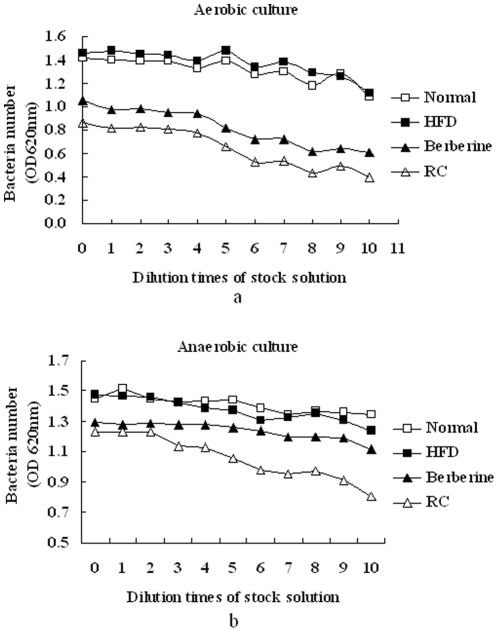
Effects of RC and berberine on growth of fecal bacteria ex vivo under (a) aerobic and (b) anaerobic conditions. “Normal”, normal chow diet-fed mice; “HFD”, high-fat diet-fed control mice; “Berberine”, Berberine-treated HFD mice; “RC”, Rhizoma Coptidis-treated HFD mice (n = 6).

After 24–26 h of culture ex vivo, no significant differences in fecal bacteria growth between HFD and normal mice were observed under both aerobic and anaerobic conditions (Fig. 3). However, RC and berberine significantly inhibited the growth of fecal bacteria under both aerobic and anaerobic conditions.

### Effect of RC and berberine on growth of Lactobacillus sp. under anaerobic conditions in vitro

**Figure 4:**
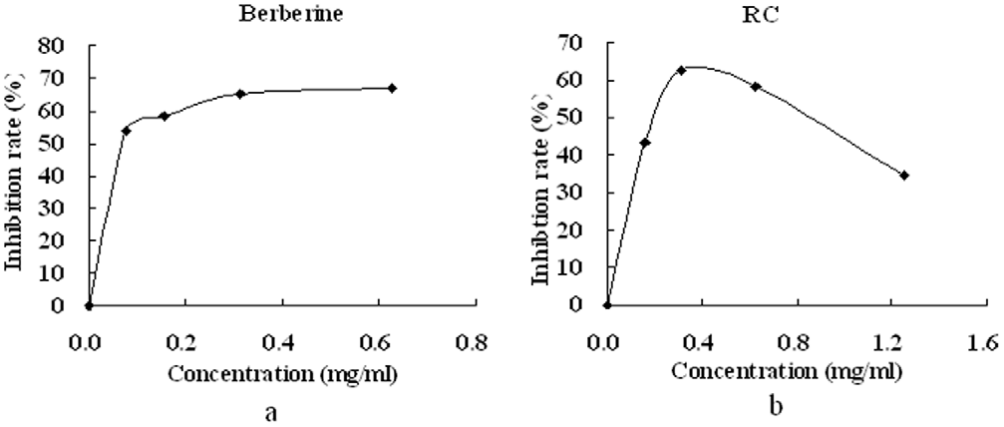
Effects of (a) berberine and (b) RC on the growth of *Lactobacillus* sp. under anaerobic conditions in vitro.

After 24–26 h of culture in vitro, RC at a minimum concentration of 0.313 mg/mL and berberine at a minimum concentration of 0.078 mg/mL significantly inhibited the growth of *Lactobacillus* sp., a classic type of Firmicutes, by more than 50% (Fig. 4). In addition, high concentrations of RC and berberine (more than 0.5 mg/mL) interfered with the assay of bacteria turbidity at 620 nm. Using Excel software, the IC_50_ values of RC and berberine were calculated to be 0.199 and 0.044 mg/mL, respectively. In the intestinal tracts of mice, the inhibition concentrations may be far less than the actual concentrations because mice were orally administrated with 200 mg/kg RC or berberine.

### Effect of RC and berberine on Fiaf protein expressions in intestinal and visceral adipose tissues

**Figure 5:**
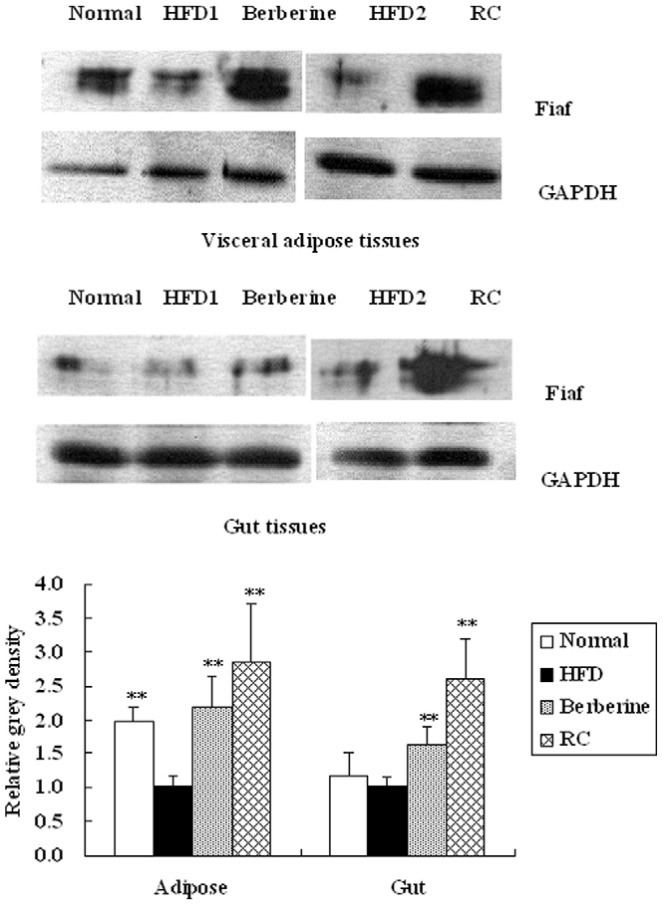
Effects of RC and berberine on Fiaf protein expressions in intestinal and adipocyte tissues. “Normal”, normal chow diet-fed mice; “HFD”, high-fat diet-fed control mice; “berberine”, berberine-treated HFD mice; “RC”, *Rhizoma coptidis*-treated HFD mice. Data were expressed as Mean±S.D. (n = 6), *P<0.05, **P<0.01 vs. HFD controls.

Fiaf expression was significantly decreased in visceral adipose tissues of HFD mice compared with normal controls (Fig. 5). However, Fiaf expression in gut tissues showed decreasing tendency in HFD mice compared with normal controls, but had no significant change. A greater number of samples may be required in future trials. Despite this limitation, both RC and berberine significantly increased the expression of Fiaf in either visceral adipose tissues or intestines.

### Effect of RC and berberine on gene expressions in visceral adipose tissues and livers

**Figure 6:**
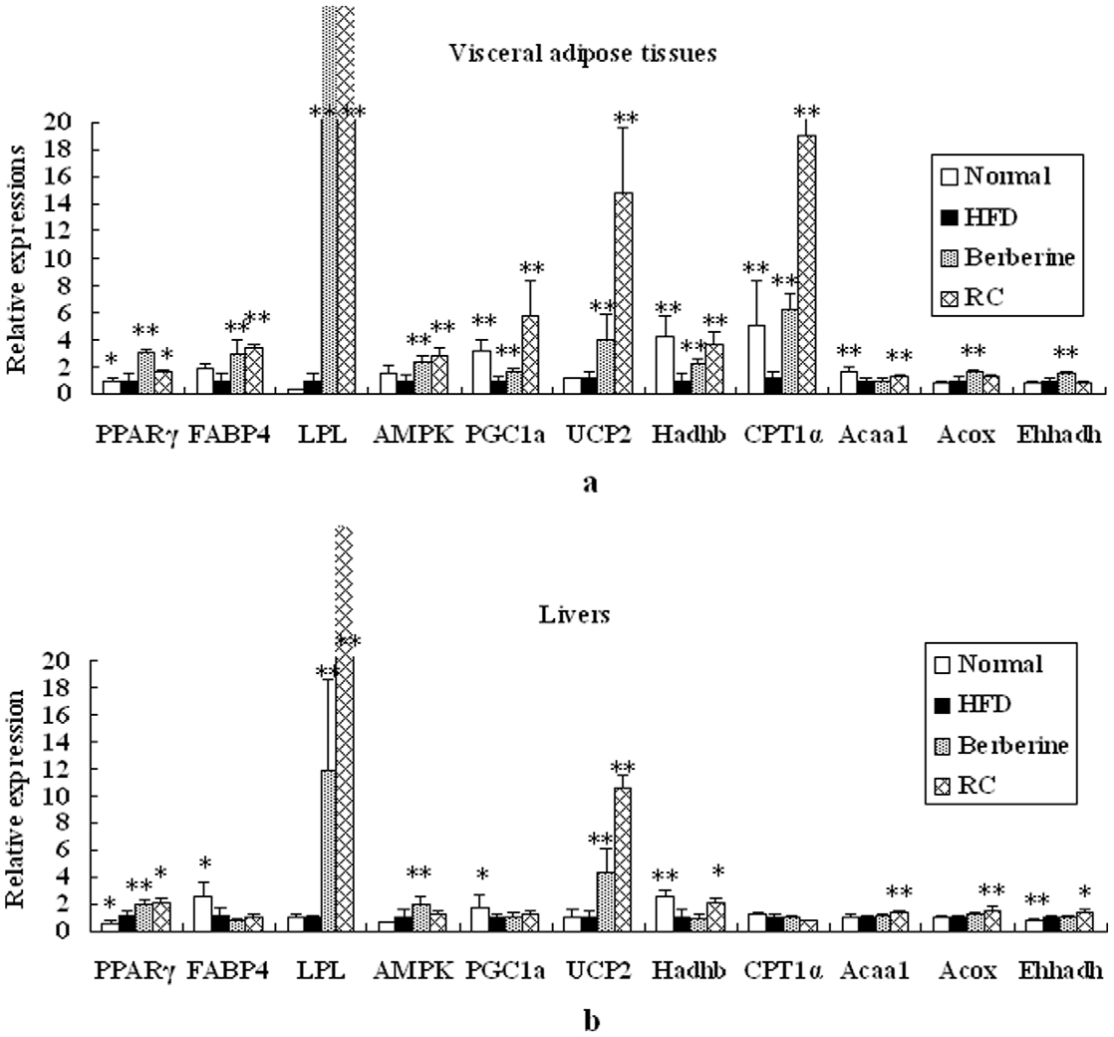
Effects of RC and berberine on gene expressions in visceral adipose tissues and livers. “Normal”, normal chow diet-fed mice; “HFD”, high-fat diet-fed control mice; “berberine”, berberine-treated HFD mice; “RC”, *Rhizoma coptidis*-treated HFD mice. Data were expressed as Mean±S.D. (n = 6), *P<0.05, **P<0.01 vs. HFD controls.

For visceral adipose tissues, mRNA expressions of PGC1α, Hadhb, CPT1α, and Acaal were significantly decreased in HFD mice compared with normal controls (Fig. 6a). Both RC and berberine significantly increased mRNA expressions of AMPK1α, PGC1α, UCP2, Hadhb, and CPT1α in HFD mice, suggesting that RC and berberine activate energy metabolism. In the present study, RC and berberine significantly activated the expressions of key genes responsible for adipogenesis, such as PPARγ2, FABP4, and LPL. In addition, RC significantly increased Acaa1 expression, whereas berberine increased the expressions of Acox and Ehhadh.

For liver tissues, mRNA expressions of PGC1α and Hadhb were significantly decreased in HFD mice compared with normal controls (Fig. 6b). RC significantly increased mRNA expressions of UCP2, Hadhb, Acaal, Acox1, and Ehhadh, whereas berberine significantly increased mRNA expressions of AMPK1α and UCP2. In the present study, both RC and berberine significantly activated the expression of key genes mediating adipogenesis, such as PPARγ2 and LPL. RC and berberine significantly increased LPL expression by more than 10-fold in either visceral adipose or liver tissues.

## Discussion

Although normal chow diets induce obesity in obesity-prone animals [19], a high-fat diet remains an important catalyst for the induction of animal models with visceral obesity [20]–[22] in a short term. A high-fat diet increases blood glucose and total cholesterol levels, although it has no significant influence in serum triglycerides as previously reported in C57BL/6 [21], NIH [22], and ICR [23] mice. High-fat diet-fed obesity may be associated with increase of calorie intake in C57BL/6 mouse model. RC and berberine significantly normalized blood glucose and lipid levels and reduced body weight gain and visceral obesity in HFD mice, suggesting that both may be directly used to treat diet-induced obesity. RC and berberine at appropriate dosages did not significantly affect dietary calorie intake and did not inhibit intestinal lipid absorption in the present study. Therefore, additional anti-obesity mechanisms should be further elucidated for both RC and berberine. Interestingly, both RC and berberine significantly inhibited the degradation of dietary polysaccharides, which might contribute to the decrease in additional calorie intake in guts or *de novo* lipogenesis.

Gut microbes, as environmental factors, regulate fat storage and metabolism [3], [24]. Gut microbiota in adult germ-free mice produce rapid increases in body fat content, which may be associated with the acceleration of the processing of dietary polysaccharides and induction of *de novo* lipogenesis. As well, gut microbiota in adult germ-free mice may be associated with decreased fatty acid oxidation. Relative proportions of microbial communities also affect body weight gain. Bacteroidetes and Firmicutes are two main communities that affect energy metabolism homeostasis [25]. Some studies suggest that obese animals or humans have more Firmicutes and less Bacteroidetes than lean controls [26], [27], and, therefore, novel therapeutic agents using probiotics, antibiotics, and/or prebiotics to treat obesity should be developed [25]. However, limited data support this idea.

*Rhizoma coptidis*, a traditional Chinese medicine, together with its active compound, berberine, has known effects against a variety of organisms. In the trial ex vivo, both RC and berberine inhibited the growth of either aerobic or anaerobic microbes. Furthermore, RC and berberine decreased the number of Firmicutes and lowered that of Bacteroidetes in the feces of HFD mice. *Lactobacillus* sp. is a classical type of Firmicutes. In the in vitro trials, specific dosages of RC and berberine significantly inhibited the growth of *Lactobacillus*, with both RC and berberine exerting hypoglycemic or hypolipidemic and body-regulating effects. In our preliminary trial, metronidazole, an antimicrobial drug against anaerobic bacteria, also significantly inhibited body weight gain (data not shown). Therefore, both RC and berberine exert anti-obesity effects by inhibiting fecal microbes. This inhibition effect might be associated with the increase in fecal polysaccharide content and decrease in intestinal calorie intake and *de novo* lipogenesis as prescribed above.

Increased expression of Fiaf in germ-free mice was selectively suppressed in the intestinal epithelium after exposure to gut microbes [24]. Conversely, RC and berberine significantly promoted the expression of Fiaf in intestinal and adipose tissues in HFD mice, which may be associated with their antimicrobial activities. HFD may affect the proportion of communities of intestinal microbes (although not in a significant manner) and regulate the expression of Fiaf. In the present study, Fiaf expression in visceral adipose tissues of HFD mice was significantly decreased compared with normal controls. Increased Fiaf expression enhances energy metabolism by activating AMPK and up-regulating PGC1α [3]. RC and berberine increase Fiaf expression and may increase energy metabolism by activating AMPK and PGC1α pathways. Activation of AMPK enhances oxidative metabolism and mitochondrial biogenesis, and then switches on catabolic pathways that produce ATP [28]. PGC1α controls many aspects of oxidative metabolism including mitochondrial biogenesis and respiration [29].

Upregulation of PGC1a or activation of AMPK promotes mitochondrial energy or lipid oxidation metabolism and causes wide and coordinated changes in the related genes. UCP2, CPT1α, and Hadhb are marker genes responsible for energy or mitochondrial fat oxidation metabolism. UCP2 is widely expressed in tissues and likely associated with the control of reactive oxygen species production by mitochondria, regulation of ATP synthesis, and fatty acid oxidation [30]. CPT1α is the rate-limiting enzyme carnitine palmitoyl-transferase, which regulates fatty acid oxidation [31] and is regulated by PGC1α. Hadhb is the mitochondrial trifunctional protein with a multienzyme complex of the fatty acid β-oxidation cycle [32]. In the present study, RC and berberine significantly promoted mRNA expressions of these energy metabolism genes in HFD mice, suggesting that both may activate mitochondrial energy or fat oxidation metabolism. This increase is mainly present in visceral adipose tissues, indicating that both RC and berberine directly activate mitochondrial energy metabolism in visceral adipose tissues.

Acox1, Acaal, and Ehhadh are key genes responsible for peroxisomal β-oxidation. Peroxisomal β-oxidation plays an important role in fat metabolism [20]. In livers or visceral adipose tissues, RC or berberine slightly or moderately increased these or part of these gene expressions responsible for peroxisomal β-oxidation. The increase was not as highly expressed as observed in mitochondrial genes, suggesting that RC or berberine mainly enhance energy metabolism through the mitochondrial, not the peroxisomal pathway.

RC and berberine increased the expressions of mRNAs responsible for adipogenesis (PPARγ2, FABP4, and LPL), which may be associated with a compensatory response to increased mitochondrial energy metabolism, because both RC and berberine actually lowered body weight and visceral fat accumulation. PPARγ2 and LPL play important roles in adipocyte differentiation [33] and fatty acid intake [34], respectively. Activation of PPARγ2 and LPL increases insulin sensitivity [35] and lowers serum triglycerides [34], respectively. In previous trials in vivo, berberine significantly increased LPL activities [36]. Taken together, these may explain why RC and berberine have anti-hyperglycemic, anti-hyperlipidemic, and anti-obesity effects.

In the present study, the effects of RC were comparable to berberine at a similar dose (200 mg/kg). Therefore, berberine is a key active compound of RC. In addition, RC only contained 24.2% berberine but the effects of RC were comparable to those of berberine at a similar dose (200 mg/kg). This finding suggests that other compounds of RC may exert an effect similar to or stronger than that of berberine. However, this may require further studies in the future. The preparation of RC is very simple and low-cost compared with berberine. No untoward effects of RC were found in the animals during the testing period. Therefore, RC shows better application potential in patients with diet-induced obesity.

In conclusion, in the present study, we first showed that both RC and berberine exert anti-hyperglycemic, antihyperlipidemic, and anti-obesity effects likely mediated by inhibiting gut microbes. The antimicrobial activities of RC and berberine may result in decreasing degradation of dietary polysaccharides (it seemed to have no influence on intestinal lipid absorption), lowering additional calorie intake (from dietary polysaccharides) and *de novo* lipogenesis, and then systemically increasing expression of Fiaf protein and promoting expressions of energy metabolism genes (AMPK1α, PGC1α, UCP2, CPT1α, and Hadhb) in visceral adipose tissues. Taken together, these activities may contribute to significant anti-obesity effects. Berberine may be a key active compound of RC that is responsible for these effects. In this study, we firstly show the evidence that pharmacological regulation of gut microbes by RC and berberine can develop an anti-obesity strategy. Future studies should focus on developing more appropriate drugs with antimicrobial activities to control diet-induced obesity.

## Materials and Methods

### Diets and animals

Four-week old male C57BL/6J mice [SPF grade, Certified No. SCXK (Hu) 2007-0005] were obtained from Shanghai Slac Laboratory Animal, Co., Ltd. (Shanghai, China). Animals were kept in an environmentally controlled breeding room (temperature: 20±2°C, humidity: 60±5%, 12 h dark/light cycle). They were fed standard laboratory chow with water *ad libitum* and fasted from 9:00 am to 3:00 pm before the experiments. The study was carried out in strict accordance with the recommendations in the Guide for the Care and Use of Laboratory Animals of Institutional Animal Care and Use Committee of Tsinghua University. The protocol was approved by the Animal Welfare and Ethics Committee of Tsinghua University, China (No. 2010-XWD-AE). All surgeries were performed under sodium pentobarbital anesthesia, and all efforts were made to minimize suffering. Both normal chow and high-fat diets were purchased from Shanghai Slac Laboratory Animal Co., Ltd. Normal chow diets contained 20.5% crude protein, 4.62% crude fat, 52.5% nitrogen-free extract, and 4.35% crude fibers (total calories 3.45 Kcal/g, 12% calories in fat). High-fat diets contained 18.8% crude protein, 16.2% crude fat, 45.2% nitrogen-free extract, and 3.98% crude fibers (total calories 3.79 Kcal/g, 38% calories in fat).

### Preparation of ethanol extracts of RC

**Figure 7:**
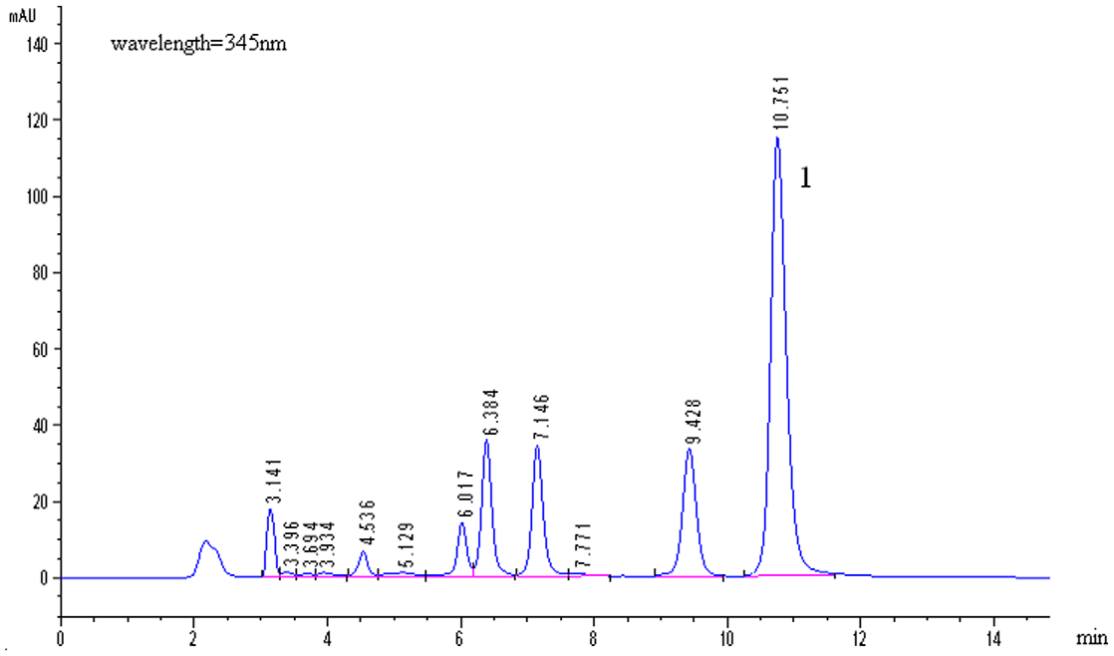
HPLC fingerprint of ethanol extracts of *Rhizoma coptidis* (RC). Chromatogram conditions: Kromasil C18 column (5 µm, 4.6 mm×250 mm), column temperature, 30°C; mobile phase, acetonitrile: H_2_O (0.05 M KH2PO4, pH 3.0) = 30∶70 (v/v); elution rate, 1 ml/min; detector wavelength, 345 nm. Peak “1”, berberine (content, 24.2%).

Ethanol extracts of RC were prepared by Dr. Qixin Yan of Neptunus Bio-Engineering Co., Ltd., Shenzhen, China. In brief, 611 g dried RC powder were refluxed with 5 L of 75% ethanol for 3 h. The extracted solution was then filtered with 3–5 mm thin cotton, and the filtered solution was dried by water bath at 90°C. The residues were dried by vacuum dryer at 50°C overnight. The yield of the ethanolic extracts of RC was 17.10% (g/g). The HPLC fingerprint of the ethanol extracts of RC is shown in Fig. 7 to guarantee stable quality control. Berberine, a potential active compound of the ethanol extracts of RC, was found to have a content of about 24.2%.

### Experimental protocol

Mice were divided into four groups: common chow diet-fed normal animals (Normal), high-fat diet-fed controls (HFD), berberine (200 mg/kg)-treated high-fat diet-fed animals, and RC (200 mg/kg)-treated high-fat diet-fed animals. RC or berberine (Guandong Huanan Pharmacy, Ltd., China) was freshly suspended in distilled water and orally administered to the mice by gavage. Identical volumes of distilled water were given to normal and HFD controls. Body weight was monitored once a week. The dietary intake, water intake, urine volume, and fecal weight of each mouse were recorded while in the metabolic cage for 24 h, once a week. Total calorie intake was calculated according to dietary calorie intake, and expressed as Kcal/mice/day. Freshly collected feces were used for lipid and bacteria number assays. After six weeks of normal and high-fat diet feeding, the animals were weighed and anesthetized by intraperitoneal injection of pentobarbital at a dose of 35 mg/kg. Blood was collected from the orbital plexus after the six-week treatment. Serum was isolated by centrifugation at 1500 g at 4°C for 10 min and stored at −80°C until it was used for blood biochemical assays. Following blood collection, the anesthetized mice were sacrificed by cervical dislocation. Visceral adipose tissues (perigonadal fat, the main part of the internal white adipose tissues), livers, and intestinal tracts were removed from the animals. Visceral adipose tissues were immediately weighed after removal from the animal. The samples were instantly frozen in liquid nitrogen and then stored at −80°C until they were used for biochemical analysis.

### Biochemical analysis

Blood glucose, total cholesterol, and triglycerides were assayed using commercial kits (BioSino Bio-Technology and Science, Inc., Beijing, China). Blood glucose was estimated by the glucose oxidase method [37]. Total cholesterol was estimated by the method of Allain et al. [38]. Triglyceride levels were determined by the enzymatic method [39]. Fecal lipid assay was conducted according to a previous protocol [19]. Fecal polysaccharide assay was according to anthrone-reagent method [40].

### 16SrRNA gene expression analysis of fecal Firmicutes and Bacteroidetes in mice by Real-time PCR

The 16SrRNA analysis of Firmicutes, Bacteroidetes, and total bacteria in feces was performed in accordance with a previous method [41] with slight modifications. Briefly, about 0.1–0.2 mg fresh fecal samples from the intestines of different groups of mice were collected and suspended with 1 mL PBS in a 1.5-mL Eppendorf tube. The fecal suspension was centrifuged at 500–1000 rpm for 5 min. The collected supernatants were further centrifuged at 10,000 rpm for 5 min. The deposit containing fecal bacteria was used to extract gnomic DNA. Genomic DNA was extracted with a TaKaRa minibest bacterial genomic DNA extraction kit (TaKaRa, Dalian, China). The DNA samples were then subjected to DNA quantitative analysis. *Clostridium perfringens* (CICC No. 22949, China Center of Industrial Culture Collection, China) was selected as standard strain for quantitative assays of the relative content of 16SrRNA gene of Firmicutes and total bacteria. *Bacteroides thetaiotaomicron* (ATCC® 29741™) served as standard strain for quantitative assays of the relative content of 16SrRNA gene of Bacteroidetes. The primers of Firmicutes, Bacteroidetes, and total bacteria were set as shown in Table 2. Data of relative expression contents were calculated according to the following steps and formulas:

*I)* 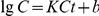

*II)* 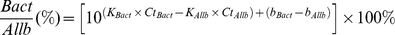

*III)* 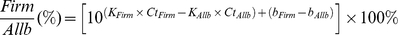

*IV)* *16SrRNA relative expression of* 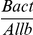 *in each group*

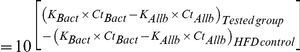

*V)* *16SrRNA relative expression of* 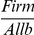 *in each group*

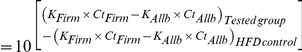
where C is the DNA copy number, K_Bact_ or K_Firm_ is the slope of the linear regression and correlation formula (lgC = KCt+b) from the standard curves of Bacteroidetes or Firmicutes controls, respectively, and Ct refers to Ct values of from the q-PCR of Bacteroidetes (Bact), Firmicutes (Firm), and total bacteria (Allb).

**Table 2:** Primers for Bacteroidetes, Firmicutes, and total bacteria.

	Primers (5′ to 3′)		Size(bp)
Bacteroidetes	**Forward**	GGARCATGTGGTTTAATTCGATGAT	126
	**Reverse**	AGCTGACGACAACCATGCAG	
Firmicutes	**Forward**	GGAGYATGTGGTTTAATTCGAAGCA	126
	**Reverse**	AGCTGACGACAACCATGCAC	
Total bacteria	**Forward**	ACTCCTACGGGAGGCAGCAG	200
	**Reverse**	ATTACCGCGGCTGCTGG	

### Fecal bacteria culture ex vivo under anaerobic or aerobic condition

As previously described, collected fecal bacteria were suspended in PBS and normalized according to fecal weight. First, 5 µ: fecal bacteria stock solution (OD620 nm 0.2–0.4) was added to 200 µL MRS Broth culture (Qingdao Hope Bio-Technology Co., Ltd, China) and diluted with 200 µL MRS Broth culture 2^1–10^ times in a 96-well plate. For anaerobic cultures, as 96-well plate containing the bacterial solution at different dilution concentrations was placed in a Pack-Rectangular Jar 2.5 Liter with one AnaeroPack sachet freshly opened (AnaeroPack System, Mitsubishi Gas Chemical Co., Inc., Japan). The Pack-Rectangular Jar 2.5 Liter was then placed in 37°C controlled incubator. After 24–36 h of culture, the culture solution containing bacteria was slightly re-suspended. Finally, the wells were assayed at 620 nm for bacteria turbidity. For aerobic culture, the 96-well plate was directly placed in a 37°C controlled incubator. The other procedures were similar to those of the anaerobic culture.

### Lactobacillus culture in vitro

First, 1 µL *Lactobacillus* (No. 21024, CICC) stock solution (OD620 nm 0.2–0.4) was added to 200 µL MRS broth culture in a 96-well plate. At the same time, RC at final concentrations of 80, 40, 20, 10, 5, 2.5, 1.25, 0.625, 0.313, 0.156, and 0 mg/mL or berberine at final concentrations of 40, 20, 10, 5, 2.5, 1.25, 0.625, 0.313, 0.156, 0.078, and 0 mg/mL was added to 200 µL MRS broth culture. Afterwards, the 96-well plate was placed in a Pack-Rectangular Jar 2.5 Liter with one freshly opened AnaeroPack sachet. The Pack-Rectangular Jar 2.5 Liter was then placed in a controlled incubator set to 37°C. After 24–36 h of culture, the culture solution containing bacteria was slightly re-suspended. Finally, the wells were assayed at 620 nm for bacteria turbidity. Inhibition rate (%) was calculated according to the formula:
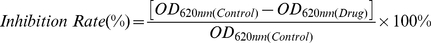



### Western blot

Freshly prepared visceral adipose tissues or small intestines (n = 6) were homogenated and lysed with NETN buffer (20 mM Tris-HCl, pH 7.8, 1 mM EDTA, 50 mM sodium chloride, and 0.5% NP-40). Lysates were centrifuged at 12,000 rpm at 4°C for 2–10 min. Supernatants were collected and protein concentration was determined by a bicinchoninic acid protein assay kit (Nanjing Jiancheng Biotech, China). Western blot analysis was carried out according to the manufacturer's protocol. Antibodies against Fiaf and GAPDH (Santa Cruz Biotechnology, Inc.) were used. Protein expression was visualized with horseradish peroxidase-conjugated secondary antibodies (Amersham Biosciences, USA; 1∶2000) and enhanced chemiluminescence (KPL, USA).

### Real-time PCR analysis of mRNAs

Approximately 100 mg of visceral adipose tissues were removed and immediately frozen in liquid nitrogen (n = 6). Total RNA was extracted using TRIZOL reagent (Invitrogen) according to themanufacturer's instructions. Reverse transcription was carried out using PrimeScript™ 1st Strand cDNA Synthesis Kit (Takara, Dalian, China). The cDNA fragments were amplified using Taq DNA Polymerase (Takara, Dalian, China) according to manufacturer's instructions. Many key genes are responsible for adipogenesis and fat oxidation metabolism. For example, peroxisome proliferators activate receptor gamma (PPARγ), fatty acid binding protein 4 (FABP4) and lipoprotein lipase (LPL) are key marker genes for adipogenesis, and AMP-activated protein kinase (AMPK), peroxisome proliferator-activated receptor gamma coactivator 1-alpha (PGC1α), uncoupling protein 2 (UCP2), and carnitine palmitoyl transferase 1-alpha (CPT1α) are key genes for lipid oxidation or mitochondrial energy metabolism. As previously described [20], there are three peroxisomal β-oxidation genes, namely, acyl-CoA oxidase 1 palmitoyl (Acox1), enoyl-CoA hydratase/3-hydroxyacyl–CoA dehydrogenase (Ehhadh), 3-oxoacyl–CoA thiolase (Acaa1), and one mitochondrial β-oxidation gene, hydroxyacyl-CoA dehydrogenase/3-ketoacyl–CoA thiolase/enoyl-CoA hydratase (Hadhb, trifunctional protein, beta subunit). In the present study, these genes were chosen for quantitative real-time PCR (q-PCR), and primers were synthesized from Invitrogen (Table 3). Glyceraldehyde-3-phosphate dehydrogenase (GAPDH) was used as internal control for normalization. q-PCR analysis was conducted by the method of SYBR® Green I dye according to the protocol of the kit (Code No. DRR081S, Takara, Dalian, China) in an ABI PRISM 7300 Real time PCR system (Applied Biosystems, USA). The q-PCR analysis involved two steps: 1) predenaturation of cDNA samples at 95°C for 30 s at the first stage, and then 2) amplification of denatured cDNA samples by 40 cycles at 95°C for 5 s and then at 60°C for 31 s at the second stage. Data were expressed as raw relative quantitation (2^−ddCt^).

**Table 3:** Primers for energy metabolism genes.

Gene names	NCBI Accession No.	Primers (5′ to 3′)	Sizes (bp)
PPARγ2	NM_011146.3	Forward: TTTCAAGGGTGCCAGTTT	152
		Reverse: GAGGCCAGCATCGTGTAG	
FABP4	NM_024406.2	Forward: AAATCACCGCAGACGACA	138
		Reverse: CACATTCCACCACCAGCT	
LPL	NM_008509.2	Forward: AACAATCTGGGCTATGA	262
		Reverse: CCACCTCCGTGTAAATC	
AMPKα1	NM_001013367.3	Forward: TTCAGGCACCCTCACATC	268
		Reverse: GACCAAAGTCGGCTATCT	
PGC1α	NM_008904.2	Forward: ACAGCAAAAGCCACAAAG	259
		Reverse: TAAGGTTCGCTCAATAGTC	
UCP2	NM_011671.4	Forward: AATGTTGCCCGTAATGCC	299
		Reverse: CCCAAGCGGAGAAAGGAA	
CPT1α	NM_013495.2	Forward: CGTGACGTTGGACGAATC	165
		Reverse: TCTGCGTTTATGCCTATC	
Acox1	NM_015729	Forward: CCGCCTATGCCTTCCACT	182
		Reverse: ACCGCAAGCCATCCGACA	
Ehhadh	NM_023737.3	Forward: TGGACCATACGGTTAGAG	213
		Reverse: CAATCCGATAGTGACAGC	
Acaa1	NM_130864.3	Forward: GATGACCTCGGAGAATGTGG	188
		Reverse: CCTGAGACACGGTGATGGT	
Hadhb	NM_145558	Forward: TGTCAGGCACTTCGTAT	153
		Reverse: TAGCCACATTGCTTGTT	
GAPDH	NM_008084	Forward: TCTCCTGCGACTTCAACA	178
		Reverse: TGGTCCAGGGTTTCTTACT	

### Statistical analysis

Data were expressed as Mean±S.D. Statistical analysis was performed using ANOVA. Newman–Keuls comparisons were used to determine the source of significant differences where appropriate. *P* values less than 0.05 were considered statistically significant.
